# Screening of FDA-Approved Drugs Using a MERS-CoV Clinical Isolate from South Korea Identifies Potential Therapeutic Options for COVID-19

**DOI:** 10.3390/v13040651

**Published:** 2021-04-09

**Authors:** Meehyun Ko, So Young Chang, Soo Young Byun, Aleksandr Ianevski, Inhee Choi, Anne-Laure Pham Hung d’Alexandry d’Orengiani, Erlend Ravlo, Wei Wang, Magnar Bjørås, Denis E. Kainov, David Shum, Ji-Young Min, Marc P. Windisch

**Affiliations:** 1 Respiratory Virus Laboratory, Emerging Virus Group, Discovery Biology Department, Institut Pasteur Korea, Seongnam 13488, Gyeonggi, Korea; meehyun.ko@ip-korea.org (M.K.); sychang0709@gmail.com (S.Y.C.); sephrene@gmail.com (A.-L.P.H.d.d.); 2 Screening Discovery Platform, Translation Research Division, Institut Pasteur Korea, Seongnam 13488, Gyeonggi, Korea; sooyoung.byun@ip-korea.org (S.Y.B.); david.shum@ip-korea.org (D.S.); 3 Department of Clinical and Molecular Medicine, Norwegian University of Science and Technology, 7491 Trondheim, Norway; aleksandr.ianevski@ntnu.no (A.I.); erlend.ravlo@ntnu.no (E.R.); wei.wang@ntnu.no (W.W.); magnar.bjoras@ntnu.no (M.B.); denis.kainov@ntnu.no (D.E.K.); 4 Medicinal Chemistry, Medicinal Chemistry & Business Development Group, Translational Research Department, Institut Pasteur Korea, Seongnam 13488, Gyeonggi, Korea; inhee.choi@ip-korea.org; 5 Institute of Technology, University of Tartu, 50090 Tartu, Estonia; 6 Institute for Molecular Medicine Finland, University of Helsinki, 00100 Helsinki, Finland; 7 Applied Molecular Virology Laboratory, Unmet Medical Needs Group, Discovery Biology Department, Institut Pasteur Korea, Seongnam 13488, Gyeonggi, Korea; 8 Division of Bio-Medical Science and Technology, University of Science and Technology, Yuseong-gu 305-350, Daejeon, Korea

**Keywords:** Middle East respiratory syndrome coronavirus, severe acute respiratory syndrome coronavirus disease, clinical isolate, high-content screening, FDA-approved drugs, drug repurposing, drug combinations, lung organoids, COVID-19, pandemic

## Abstract

Therapeutic options for coronaviruses remain limited. To address this unmet medical need, we screened 5406 compounds, including United States Food and Drug Administration (FDA)-approved drugs and bioactives, for activity against a South Korean Middle East respiratory syndrome coronavirus (MERS-CoV) clinical isolate. Among 221 identified hits, 54 had therapeutic indexes (TI) greater than 6, representing effective drugs. The time-of-addition studies with selected drugs demonstrated eight and four FDA-approved drugs which acted on the early and late stages of the viral life cycle, respectively. Confirmed hits included several cardiotonic agents (TI > 100), atovaquone, an anti-malarial (TI > 34), and ciclesonide, an inhalable corticosteroid (TI > 6). Furthermore, utilizing the severe acute respiratory syndrome coronavirus 2 (SARS-CoV-2), we tested combinations of remdesivir with selected drugs in Vero-E6 and Calu-3 cells, in lung organoids, and identified ciclesonide, nelfinavir, and camostat to be at least additive in vitro. Our results identify potential therapeutic options for MERS-CoV infections, and provide a basis to treat coronavirus disease 2019 (COVID-19) and other coronavirus-related illnesses.

## 1. Introduction

Coronaviruses (CoVs) are enveloped, positive-sense, single-stranded RNA viruses in the Coronaviridae family of the Nidovirales order. CoVs usually cause mild to severe respiratory tract infections [1]. The two types of human coronaviruses that had been described prior to 2003, coronavirus 229E and OC43, caused mild, cold-like symptoms [2,3]. However, an outbreak of severe acute respiratory syndrome (SARS) in 2003, which occurred mainly in Southeast Asia, was attributed to a coronavirus. The outbreak resulted in 8096 confirmed cases and 774 deaths (fatality rate of 9.6%) [4].

In 2012, the novel coronavirus Human Coronavirus—Erasmus Medical Center (HCoV-EMC) was isolated from a patient in Saudi Arabia who developed pneumonia and renal failure [5]. From the first outbreak in 2012 until January 2019, the HCoV-EMC epidemic resulted in 2449 laboratory-confirmed cases and at least 845 deaths (fatality rate 34%), mainly in the Arabian Peninsula. Thus, HCoV-EMC was renamed Middle East respiratory syndrome coronavirus (MERS-CoV) [6]. Another major outbreak of MERS-CoV infection, the largest outside the Arabian Peninsula, occurred in South Korea in 2015 [7,8]. Notably, aside from the index case of MERS-CoV, the majority of viral transmissions in South Korea were nosocomial, with 186 confirmed cases across 16 clinics [7,9]. Furthermore, the World Health Organization (WHO) has reported continual waves of MERS outbreaks in the Middle East, although they have been smaller than the major 2014 outbreak [6].

Due to the severity of MERS infection and the urgent need for effective treatment, several approaches for therapeutic development have been attempted [10]. In clinical studies, a combination of ribavirin and interferon-alpha (IFN-α) therapy improved patient survival rates when administered early after the onset of infection, but had no significant effect in the late stage of infection [11,12,13]. These results suggest that broad-spectrum antivirals can be effective in MERS patients at some stages of infection, but for complete antiviral activity, a treatment specific for MERS-CoV may be required. 

Since the first identified case of the severe acute respiratory syndrome CoV-2 in Wuhan, China, in late 2019, the coronavirus disease 2019 (COVID-19) rapidly spread worldwide. The ongoing COVID-19 pandemic has already caused countless human casualties and significant socio-economic losses globally. With more than 95 million COVID-19 cases confirmed and over 2.8 million related fatalities reported (6 April 2021), there is a worldwide effort to control the spread of this devastating virus. Unfortunately, there are no CoV-specific drugs approved by the United States Food and Drug Administration (FDA) for clinical use, and the number of repurposed drugs to efficiently treat COVID-19 patients are limited. Together, we screened FDA-approved drugs using patient-derived MERS-CoV, triaged hits to discriminate between early and late viral life cycle inhibitors, confirmed selected drugs using severe acute respiratory syndrome coronavirus 2 (SARS-CoV-2), and demonstrated the added value of selected medications in combination with remdesivir.

## 2. Materials and Methods

### 2.1. Viruses, Cell Lines, and Lung Organoids

The Korean strain of MERS-CoV (MERS-CoV/KOR/KNIH/002_05_2015; MERS/KOR/2015, Genbank accession no. KT029139.1) [14] was kindly provided by Sung Soon Kim, from the Division of Respiratory Viruses, Center for Infectious Diseases, Korea National Institute of Health (KNIH), Korea Centers for Disease Control and Prevention (KCDC), and propagated in Vero cells, as previously described [15]. The isolation of SARS-CoV-2 (hCoV-19/Norway/Trondheim-S15/2020), and engineering of recombinant mCherry-expressing SARS-CoV-2 strains (SARS-CoV-2-mCherry) have been described previously [16]. Viral titers were determined by plaque assays in Vero and Vero-E6 cells as described [17]. All experiments using MERS-CoV were performed at Institut Pasteur Korea in compliance with the guidelines of the KNIH using enhanced Biosafety Level 3 (BSL-3) containment procedures in laboratories approved for use by the KCDC. The SARS-CoV-2 hCoV-19/Norway/Trondheim-S15/2020 strain has been described in a previous study. All experiments using SARS-CoV-2 and SARS-CoV-2 mCherry were performed in the BSL-3 laboratory at the Norwegian University of Science and Technology (NTNU).

Vero and Vero-E6 cells (ATCC CCL-81 and ATCC CRL-1586; Manassas, VA, USA) were maintained at 37 °C with 5% CO_2_ in Dulbecco’s modified Eagle’s medium (Welgene, Gyeongsan, Korea) supplemented with 10% heat-inactivated fetal bovine serum and 1× antibiotic–antimycotic solution (Gibco/Thermo Fisher Scientific, Waltham, MA, USA). Calu-3 cells (ATCC HTB-55; Manassas, VA, USA) were maintained at 37 °C with 5% CO_2_ in Eagle’s minimum essential medium (ATCC, Manassas, VA, USA) with 10% heat-inactivated fetal bovine serum and 1× antibiotic–antimycotic solution (Gibco/Thermo Fisher Scientific, Waltham, MA, USA). 

The lung organoids (LOs) were generated as described previously [18]. Briefly, induced pluripotent stem cells (IPSCs) were subjected to embryoid body induction using embryoid bodies (EB)/primitive streak media (10 μM Y-27632 and 3 ng/mL BMP4 in serum-free differentiation (SFD) media consisting of 375 mL Iscove’s Modified Dulbecco’s Medium (IMDM), 100 mL Ham’s F-12, 2.5 mL N2, 5 mL B27, 3.75 mL 7.5% BSA, 5 mL 1% penicillin–streptomycin, 5 mL GlutaMax, 50 μg/mL ascorbic acid, and 0.4 μM monothioglycerol) in ultra-low attachment plates, with the media being replaced with endoderm induction media (10 μM Y-27632, 0.5 ng/mL BMP4, 2.5 ng/mL FGF2, and 100 ng/mL Activin A in SFD media) the morning after. Extra media was added every day for 3 days. The embryoid bodies were collected and dissociated using 0.05% Trypsin/EDTA and plated on fibronectin-coated plates with a cell density of 85,000 cells/cm^2^. Cells were then incubated in anteriorization media-1 (100 ng/mL Noggin, and 10 μM SB431542 in SFD media), followed by an incubation with anteriorization media-2 (10 μM SB431542, and 1 μM IWP2 in SFD media). The anteriorization media-2 was replaced with ventralization media (3 μM CHIR99021, 10 ng/mL FGF10, 10 ng/mL FGF7, 10 ng/mL BMP4, and 50 nM all-trans Retinoic acid in SFD media) and incubated for two days. The cell monolayer was then lifted by gentle pipetting, and the suspended cells were transferred to an ultra-low attachment plate where they would form the lung organoids.

### 2.2. Compound Libraries

A compound library of 5406 compounds composed of FDA-approved drugs, which covers approximately 60% of all FDA-approved compounds, bioactives, kinase inhibitors, and natural products, was compiled (LOPAC, Prestwick, Microsource, Selleck, Tocris) and used for this screen. Compounds were dissolved in DMSO at 10 mM and stored at −80 °C until use.

### 2.3. Image-Based Screening and Assay Validation

Vero cells were seeded at 1.2 × 104 cells per well in Opti-PRO™ serum-free medium (SFM) supplemented with 4 mM L-glutamine and 1× antibiotic–antimycotic solution (Gibco/Thermo Fisher Scientific) in black, 384-well, μClear plates (Greiner Bio-One, Kremsmünster, Austria) at 24 h prior to the experiment. Subsequently, compounds were added to each well using an automated liquid handling system (Apricot Designs, Covina, CA, USA) before virus infection. The final concentrations of each compound were 10 μM, and the DMSO concentration was kept at 0.5% or lower. For viral infection, the plates were transferred into the BL-3 containment facility to add MERS-CoV at a multiplicity of infection (MOI) of 0.0625, and cells were fixed at 24 h post-infection (hpi) with 4% paraformaldehyd (PFA) followed by immunofluorescence analyses. MERS-CoV infection was detected using rabbit anti-MERS-CoV S antibodies, and cell viability was evaluated by Hoechst 33342 staining. Images were acquired by a Perkin Elmer Operetta (20×; Waltham, MA, USA) and analyzed by in-house developed Image Mining 3.0 (IM 3.0) plug-in software. To validate the assay, dose–response curves (DRCs) with compounds with known antiviral activities against MERS-CoV were assessed: chloroquine (CQ), and cyclosporine A (CsA) [19,20]. Compounds with >70% MERS-CoV inhibition and >70% viability were subjected to DRC analyses, as described below.

### 2.4. Dose–Response Curve Drug Analysis

The primary hits (256 hits) were used to generate 10-point DRCs, with compound concentrations from 0.05 to 25 μM. The acquired images were analyzed using in-house software to quantify cell numbers and infection ratios. The antiviral activity was normalized to positive (mock) and negative (0.5% DMSO) controls in each assay plate. DRCs were fitted by sigmoidal dose–response models, and the equation was described as Y = Bottom + (Top–Bottom)/(1 + (IC_50_/X)Hillslope) using XLfit 4 Software or Prism7. The IC_50_ was calculated from the normalized activity data set fitted curve. All IC_50_ and CC_50_ values were measured in duplicate, and the quality of each assay was controlled by Z′-factor and the coefficient of variation in percent (%CV).

### 2.5. Pharmacological Action Clustering

The information regarding the pharmacological actions of each compound was compiled by using ChemIDPlus and MeSH databases [21,22] and information provided by the vendors. Once relevant information was collected, pharmacological actions were manually reassessed to finally categorize all compounds into 43 different pharmacological actions. The information on the approval status for drugs was retrieved from DrugBank, version 5.0.7 [23].

### 2.6. Drug Combination Studies

Vero-E6 or Calu-3 cells were treated with different concentrations of two drugs and infected with SARS-CoV-2 (MOI 0.1), SARS-CoV-2-mCherry (MOI 0.1) or mock. No compounds were added to the control wells. At 72 hpi, cell viability and mCherry fluorescence was measured using CellTiter-Glo assay (Promega, Madison, WI, USA) and a PerkinElmer Victor X3 Reader. A SynergyFinder v2 web application was utilized for drug combination analysis [24]. Briefly, to quantify the degree of synergy/antagonism, the observed responses were compared to the expected combination responses, calculated based on the zero interaction potency (ZIP) reference model that assumed no interaction between drugs. Synergy scores, which represent an averaged percentage excess effect due to interactions between drugs, were quantified, with positive and negative values denoting synergy and antagonism, respectively. Furthermore, the cytotoxicity of each drug combination was subtracted. Combinations with scores >10 are considered synergistic, scores between −10 and 10 additive, and below −10 are antagonistic. LOs were treated with 0.5 μM camostat, 0.5 μM nelfinavir, 0.5 μM cepharanthine, 0.5 μM ciclesonide alone or in combinations with 0.5 μM remdesivir and infected with SARS-CoV-2-mCherry (MOI 0.1). No compounds were added to the control wells. At 72 hpi, the dead cells were stained using Cell Toxicity Green Assay (CTxG, Promega), and nuclei were stained with DAPI. Cells were fixed with PFA and imaged using microscopy. Representative images (*n* = 3) were selected.

## 3. Results

**Figure 1:**
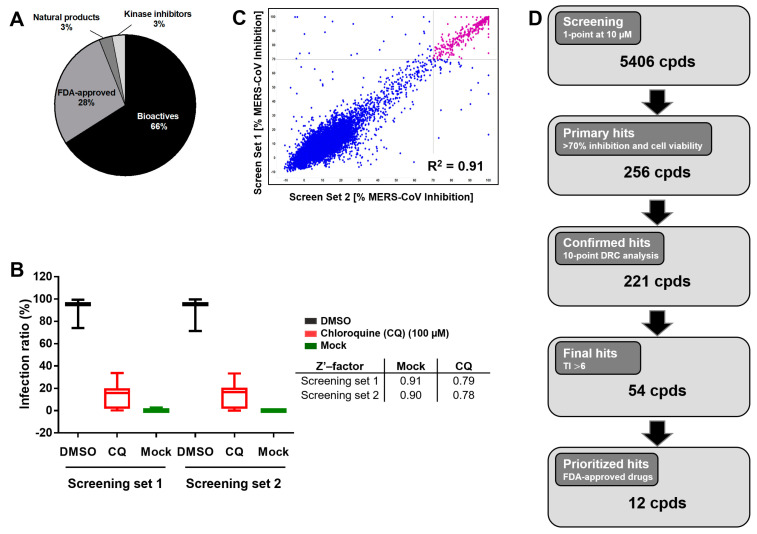
Overview of the library composition and triage of hits. (**A**) Our small-molecule compound library primarily comprised bioactives and FDA-approved drugs, with a small proportion of natural products and kinase inhibitors. (**B**) High-content screening (HCS) of 5406 compounds (cpds) in two batches in duplicate, and calculation of Z’-factors between high (MERS-CoV infection, black) and low (mock, green) values. Chloroquine (CQ). (**C**) Correlation between duplicate screens. The scatter plot shows Middle East respiratory syndrome coronavirus (MERS-CoV) inhibition ratios overlaid with cell viability ratios. Compounds with MERS-CoV inhibition >70% and cell viability >70% were regarded as primary hits. (**D**) Flowchart of HCS hit selection and confirmation of final hit selection.

To address the urgent unmet need to develop effective treatments for CoV patients, we implemented a high-content screening (HCS) strategy with the goal of repurposing newly identified MERS-CoV inhibitors for a wider range of CoVs, including COVID-19. Utilizing a Korean MERS-CoV patient isolate, we screened 5406 compounds, including FDA-approved drugs, bioactive agents, kinase inhibitors, and natural products. Our library included 60% of all FDA-approved drugs (1247 out of 2069 total) (Figure 1A). Compounds were tested for activity against MERS-CoV by analyzing the levels of expression of viral spike (S) protein in infected Vero cells using immunofluorescence analysis (IFA). The screens included the reference inhibitor chloroquine (IC_90_ = 93 μM) at 100 μM to define maximum inhibition (De Wilde et al., 2014). The calculated Z’-factor above 0.78 indicated good discrimination between the control dimethyl sulfoxide (DMSO) and chloroquine treatment of infected cells (Figure 1B). Two independent HCS analyses (screen 1 and screen 2) were conducted, demonstrating a high degree of correlation (R^2^ = 0.91) between the two replicates (Figure 1C). These screens identified 256 compounds that demonstrated >70% MERS-CoV inhibition at non-cytotoxic concentrations (>70% cell viability) (Figure 1D). These primary hits were then confirmed using a 10-point dose–response curve (DRC) analysis to determine the IC_50_ and 50% cytotoxicity concentrations (CC_50_) for each compound (Figure 1D). A representative DRC analysis is shown in Supplementary Figure S1. The therapeutic indexes (TIs) were calculated as the ratio of CC_50_/IC_50_. Among the 256 initial hits, 35 compounds were denoted as inactive (TI values < 1), and were eliminated from the list of confirmed hits. Of the resulting 221 confirmed hits, 54 compounds with an in vitro TIs > 6 were selected for further testing (Figure 1D).

**Figure 2:**
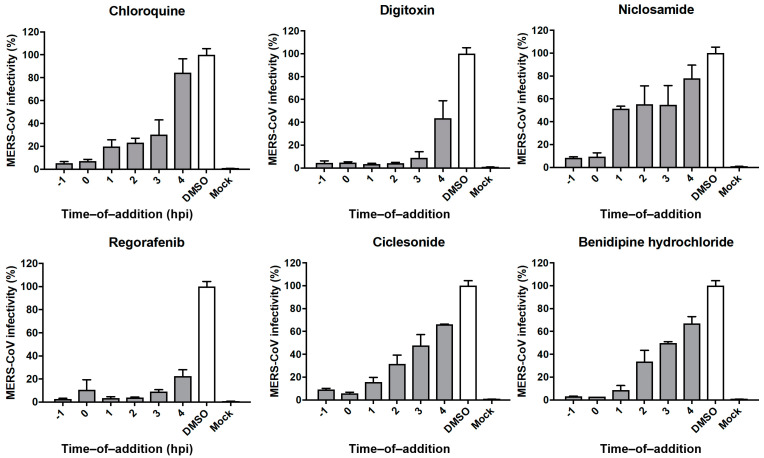
Time-of-addition study with selected FDA-approved drugs. Five FDA-approved drugs were analyzed by time-course experiments to determine the stage of the MERS-CoV life cycle inhibited. Vero cells were infected with MERS-CoV at a multiplicity of infection of 5, and FDA-approved drugs were administered at six time points pre- or post-infection as indicated. Drugs were used at concentrations above their 90% inhibitory concentration (IC_90_) values. Chloroquine served as a known early stage inhibitor.

To investigate whether the FDA-approved drugs act on the early or late stages of the viral life cycle (pre- or post-entry), we conducted time-of-addition studies. Vero cells were treated with each drug at a concentration above its IC_90_ and analyzed as described in the Supplementary Information. Chloroquine served as an early-stage inhibitor control, and inhibited MERS-CoV infection by up to 30% until 3 hpi. However, chloroquine had no significant effect when administered at 4 hpi (Figure 2). A similar outcome was observed for treatment with ouabain, digitoxin, digoxin, niclosamide, regorafenib, nelfinavir mesylate, ciclesonide, and benidipine hydrochloride, all of which inhibited MERS-CoV infection only when administered earlier than 4 hpi (Figure 2, Supplementary Figure S2). In contrast, atovaquone, lercanidipine hydrochloride, permethrin, and octocrylene had only minor inhibitory effects throughout the time-course experiments (Supplementary Figure S2).

Remdesivir, a broad-spectrum antiviral drug interfering with the RNA-dependent RNA polymerase (RdRp) activity of various RNA viruses, was approved for the treatment of SARS-CoV-2 infection. However, remdesivir alone does not prevent the infection but shortens hospitalization if administered early after infection [25,26]. Therefore, we tested remdesivir in combination with nelfinavir, ciclesonide, camostat, and cepharanthine in SARS-CoV-2- and mock-infected Vero-E6 cells, and evaluated the virus-mediated cytotoxicity by determining the ATP level. 

**Figure 3:**
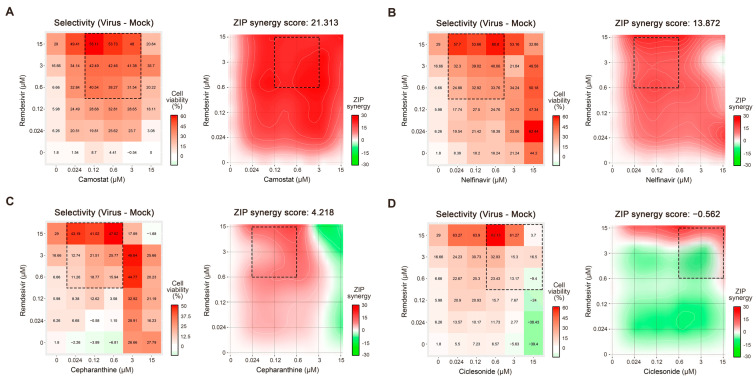
Evaluation of drug combinations in severe acute respiratory syndrome coronavirus 2 (SARS-CoV-2)-infected Vero-E6 cells. As a read-out, virus-mediated cell death in the presence and absence of drugs was assessed. (**A**) Remdesivir–camostat; (**B**) remdesivir–nelfinavir; (**C**) remdesivir–cepharanthine; and (**D**) remdesivir–ciclesonide interactions were monitored. Dose–response matrices and synergy distribution maps are shown on the right and left panels with corresponding cell viability and zero interaction potency (ZIP) synergy, respectively. X and Y axes indicate drug concentrations (μM). ZIP synergy scores were calculated as described in the Material and Methods section.

Each drug combination was tested in a 6 × 6 dose–response matrix, where five doses of single drugs were combined in a pairwise manner. We subtracted the drug combination responses measured on virus-infected cells from those measured on mock-infected cells. As a result, we obtained dose–response matrices demonstrating the selective virus inhibition achieved by each combination. We calculated the ZIP synergy scores for the whole 6 × 6 dose–response matrices and the most synergistic 3 × 3 dose regions for each drug combination. The scores show the combined virus inhibition effect beyond the effect expected from single drugs. Thereby, we observed synergistic effects for remdesivir–camostat, remdesivir–nelfinavir, and remdesivir–cepharanthine combinations (most synergistic area scores >10), and additive effects for remdesivir–ciclesonide in Vero-E6 cells (most synergistic area score between 0 and 10, Figure 3; Table 1).

**Figure 4:**
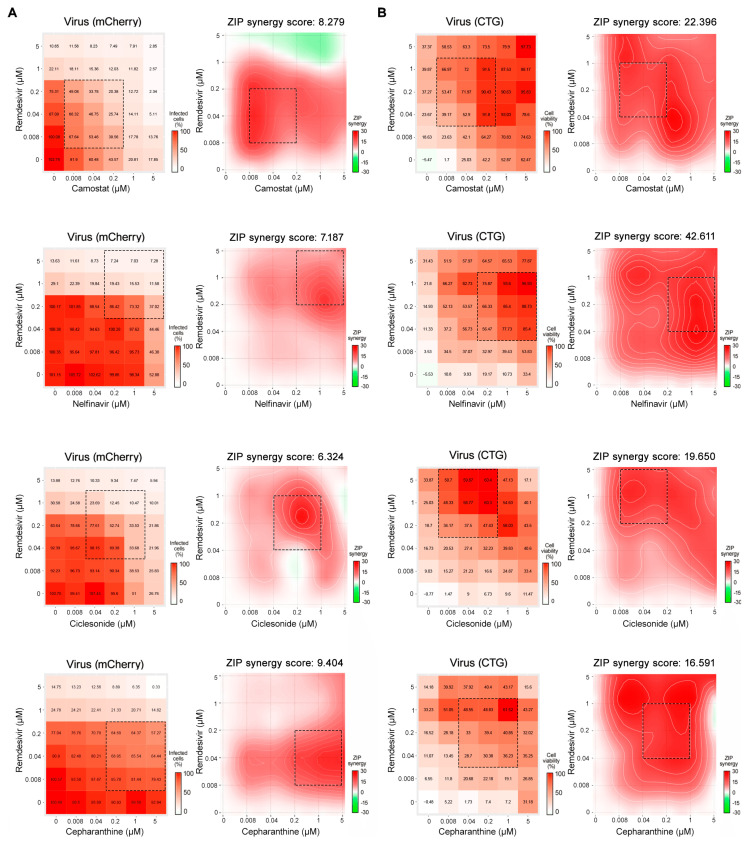
Evaluation of drug combinations in SARS-CoV-2-mCherry-infected Calu-3 cells. (**A**) The 6 × 6 dose–response matrices and interaction landscapes of remdesivir–camostat; remdesivir–nelfinavir; remdesivir–cepharanthine; and remdesivir–ciclesonide obtained using fluorescence analysis of SARS-CoV-2-mCherry-infected Calu-3 cells. ZIP synergy scores were calculated for indicated drug combinations. (**B**) The 6 × 6 dose–response matrices and interaction landscapes of remdesivir–camostat; remdesivir–nelfinavir; remdesivir–cepharanthine; and remdesivir–ciclesonide obtained using a cell viability assay (CTG) on mock-, and SARS-CoV-2-mCherry-infected Calu-3 cells. The selectivity for the indicated drug concentrations was calculated (selectivity = efficacy-(100-Toxicity)). ZIP synergy scores were calculated for indicated drug combinations.

**Figure 5:**
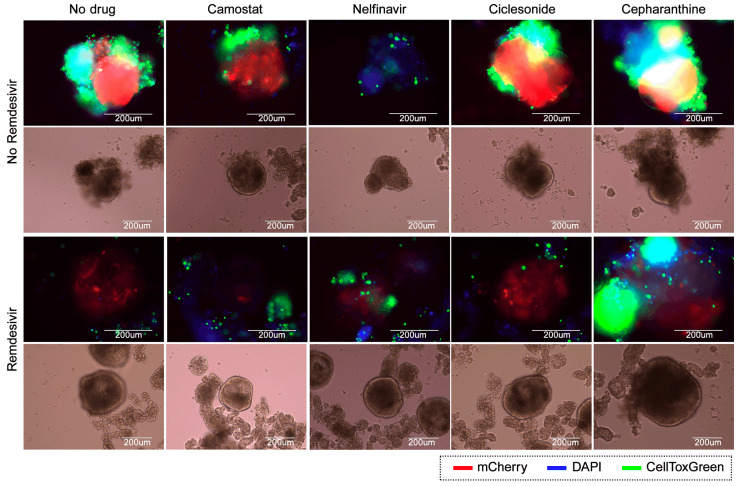
Evaluation of antiviral effects of drug combinations in human lung organoids (LOs). Fluorescent and bright-field analysis of drug or carrier-treated SARS-CoV-2-mCherry-infected LOs at 72 hpi (multiplicity of infection (MOI) 0.1). Virus infection, cell nuclei, and cytotoxicity are shown in red, blue, and green, respectively. Scale bars, 200 μm.

Then, we tested the antiviral efficacy of four combinations in Calu-3 cells using the SARS-CoV-2-mCherry virus (Figure 4) [16]. We monitored the virus-mediated expression of reporter protein and viability of virus- and mock-infected cells. Each drug combination was tested in a 6 × 6 dose–response matrix, where five doses of single drugs were combined in a pairwise manner. As a result, we obtained dose–response matrices demonstrating virus inhibition and cell viability achieved by each combination (Figure 5). We plotted synergy distribution maps, showing synergy at each pairwise dose. For each drug combination, we calculated ZIP synergy scores for the whole 6×6 dose–response matrices and for most synergistic 3 × 3 dose regions (Table 2). We observed that all combinations were synergistic based on fluorescent intensity and cell viability analyses (most synergistic area scores >10). This high synergy allowed us to substantially decrease the concentration of both components to achieve antiviral efficacy that was comparable to those of individual drugs at high concentrations.

To further evaluate the effects of drug combinations, we used IPSC-derived LOs. Fifty day-old LOs were treated with 0.5 μM camostat; 0.5 μM nelfinavir; 0.5 μM ciclesonide; 0.5 μM cepharanthine, or their combinations with 0.5 μM remdesivir, followed by infection with SARS-CoV-2-mCherry. At 72 hpi, the organoids were analyzed for viral reporter protein expression (mCherry) and cell death (CellToxGreen). Remdesivir–camostat and remdesivir–nelfinavir combinations substantially attenuated virus-mediated mCherry expression. Thus, these drug combinations should be further investigated in vitro and in vivo.

## 4. Discussion

Our approach aimed to identify FDA-approved drugs and bioactives that could be promptly repurposed or developed, respectively, to treat MERS- and potentially COVID-19-infected patients. In previously reported studies, small molecule libraries that were screened against MERS-CoV included approximately 300 drugs with FDA approval or that were in clinical development [20,27]. Our screen included 1247 FDA-approved drugs, and as a result, we identified the drugs not found in previous studies, indicating that further opportunities exist for identifying novel anti-CoV drugs by screening larger libraries of FDA-approved drugs and bioactives. Moreover, despite having used a different viral isolate than in earlier reports, we corroborated four previously identified hits, including emetine dihydrochloride, ouabain, cycloheximide, and nelfinavir mesylate. This strongly suggests that the drugs reproducibly identified in our HCS assays and in the previously published screens could be repurposed as potential therapeutic options for patients suffering from CoV infections [27].

**Figure 6:**
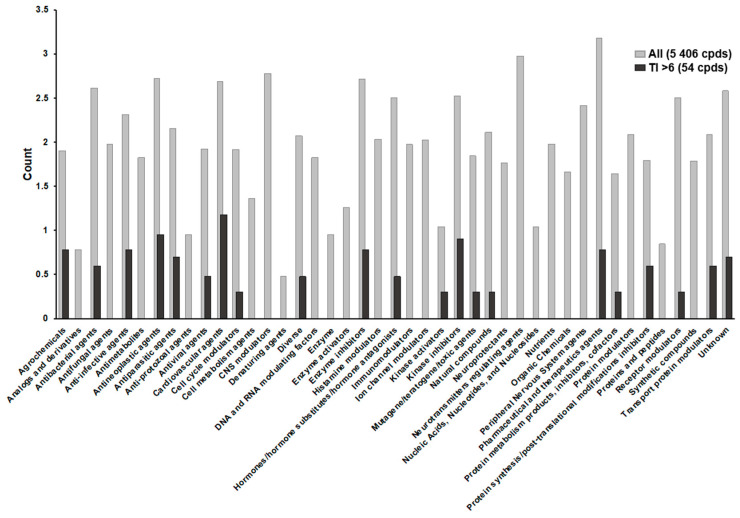
Pharmacological action profiling of all library compounds and confirmed hits. The 54 final hits were sorted into 43 pharmacological action categories. Gray and black bars indicate the distribution of all screened compounds and confirmed hits with a therapeutic index (TI) >6. The vertical axis displays counts of each compound on a log scale.

Figure 6 shows the classification of library compounds into 43 categories of pharmacological action, according to publicly available drug databases. Notably, the cardiovascular agents’ category contained 14 of the 54 final hit compounds (26%). These belong to a class of cardiac glycosides, naturally derived agents that are used for treating cardiac abnormalities and modulating sodium–potassium pump action [28]. Glycosides have also been reported to exhibit antiviral activity against the herpes simplex virus and human cytomegalovirus [29,30]. Consistent with these previous studies, our data indicate that the cardiac glycosides ouabain, digitoxin, and digoxin also efficiently inhibit MERS-CoV infection. Ouabain has been found to block cellular entry by CoV, such as MERS-CoV, through Src kinase signaling [31]. Based on these data, we speculate that cardiac glycosides may exert anti-MERS-CoV activity through the blockade of viral entry. However, more experimental work will be required to elucidate the exact mechanism by which this occurs.

Drug development may be hastened by repurposing FDA-approved drugs and inhibitors with known biological functions, pharmacological activities, and safety profiles. Therefore, we prioritized 12 FDA-approved drugs and six bioactives not yet reported to have anti-CoV activities; their information is summarized in Table 3 and Table 4, respectively. Important to note, a follow-up study confirmed seven of the 12 FDA-approved drugs listed in Table 1 as active against SARS-CoV-2 [32]. An additional 26 inhibitors that our HCS identified include bioactives and drugs that have been studied in clinical trials. A ranking of these inhibitors according to selectivity index (SI) values, ranging from >6 to >156, is shown in Supplementary Table S1.

Our time-of-addition studies demonstrated chloroquine to be effective against MERS-CoV only if administered no later than 3 hpi (Figure 2), and this was also the case for ouabain, digitoxin, digoxin, niclosamide, regorafenib, nelfinavir mesylate, ciclesonide, and benidipine hydrochloride (Figure 2, Supplementary Figure S2). Important to note, ciclesonide, an immune system suppressor used to treat asthma and allergic rhinitis, was recently shown to inhibit SARS-CoV-2 [33], the cause of COVID-19, and was reported by Japanese medical doctors to have improved pneumonia symptoms in multiple COVID-19 patients [34]. Our data are consistent with previous reports, which indicated that ouabain and other cardiotonic steroids effectively block clathrin-mediated CoV endocytosis [10,31]. In contrast, the minor inhibitory effects we observed for atovaquone, lercanidipine hydrochloride, permethrin, and octocrylene throughout the time-course indicated that these drugs likely act at later stages of the viral life cycle (Supplementary Figure S2). Notably, our results indicate that lercanidipine hydrochloride and benidipine hydrochloride, both dihydropyridine calcium channel blockers, display different patterns of viral inhibition [35,36]. This observation could be explained by the different channel selectivity of the two drugs: benidipine hydrochloride blocks triple voltage-gated calcium channels, whereas lercanidipine hydrochloride blocks single voltage-gated channels [37,38,39]. A dendrogram showing the structural relationship of 36 selected inhibitors with anti-MERS-CoV activity is shown in Supplementary Figure S3.

Combination therapies have become a standard for the treatment of human immunodeficiency virus (HIV) and hepatitis C virus (HCV) infections. They are advantageous over monotherapies due to better antiviral efficacy, reduced toxicity, as well as the ability to prevent the development of viral drug resistance, etc. In this manuscript, we demonstrated that combinations of remdesivir with nelfinavir or camostat have synergistic anti-SARS-CoV-2 effects in Vero-E6 and Calu-3 cells and lung organoids. Of note, camostat inhibits serine proteases such as the transmembrane protease serine 2 (TMPRSS2), which is not present in Vero-E6 cells. However, according to https://go.drugbank.com/drugs/DB13729 (5 April 2021), camostat targets Trypsin-1, Suppressor of tumorigenicity 14 protein, and cholecystokinin, suggesting that one of these targets might be essential for replicating coronaviruses in Vero cells. Furthermore, a phase III clinical trial has been initiated recently with remdesivir–camostat combination in Korea (5 April 2021; http://www.koreaherald.com/view.php?ud=20210104000816). Therefore, our identified drugs, in combination with remdesivir, could potentially reduce the viral load and consequently lower the probability of virus spread. 

In summary, we identified 12 FDA-approved drugs that could be repurposed for MERS-CoV or potentially COVID-19 therapy in alone or in combination with other drugs. However, further in vitro studies are needed to investigate their exact antiviral mechanisms and determine their potential synergistic effects to prioritize and select drugs for potential use in randomized, double-blind clinical trials mandatory to assess their safe use in humans.

## Figures and Tables

**Table 1:** ZIP synergy scores of drug combinations in SARS-CoV-2 infected Vero-E6 cells.

Drug Combination	Synergy Score	Most Synergistic Area Score
Remdesivir–camostat	21.3	25.7
Remdesivir–nelfinavir	13.9	19.5
Remdesivir–cepharathine	4.2	12.7
Remdesivir–ciclesonide	−0.6	6.8

**Table 2:** ZIP synergy scores of drug combinations in SARS-CoV-2-mCherry-infected Calu-3 cells.

Drug Combination	mCherry Fluorescence		CTG Assay	
	Synergy Score	Most Synergistic Area Score	Synergy Score	Most Synergistic Area Score
Remdesivir–camostat	8.3	17.3	22.4	27.7
Remdesivir–nelfinavir	7.2	11.1	42.6	51.8
Remdesivir–cepharathine	9.4	16.1	16.6	24.5
Remdesivir–ciclesonide	6.3	11.6	19.7	25.8

**Table 3:** Hit profiling and anti-MERS-CoV efficacies of FDA-approved drugs in Vero cells ^1^.

Drug Name	Trade Name	Putative Drug Target	Pharmaceutical Action	IC_50_^2^ (μM)	SD ^3^ (±)	CC_50_^4^ (μM)	TI ^5^
Ouabain ^#,†^	Strodival	Na, K-exchanging ATPase pump	Cardiotonic agent	0.08	0.0066	>25 ^§^	>312.5
Digitoxin ^#,†^	Digitaline	Ca, Na-exchanging ATPase pump	Cardiotonic agent	0.16	0.0003	>25 ^§^	>156.3
Digoxin ^#,†^	Lanoxin	Ca, Na-exchanging ATPase pump	Cardiotonic agent	0.17	0.0084	>25 ^§^	>147.1
Niclosamide ^#,†^	Niclocide, others	ATP synthase	Agrochemical	0.55	0.363	>25 ^§^	>45.5
Atovaquone *	Mepron	Unknown (lipophilic)	Anti-infective agent	0.72	0.0585	>25	>34.7
Regorafenib ^#,†^ (Bay 73–4506)	Stivarga	Multiple kinases	Anti-neoplastic agent	2.31	0.0834	>25	>10.8
Lercanidipine hydrochloride *	Zanidip	Calcium channel blocker	Cardiovascular agent	2.36	0.1654	>25	>10.6
Permethrin *	Elimite, others	Na channel	Agrochemical	3.60	0.7573	>25	>6.9
Octocrylene *	None	Estrogen receptor alpha	Additive in sunscreen	3.62	0.6435	>25	>6.9
Nelfinavir mesylate ^#,†^	Viracept	HIV-1 protease	Antiviral agent	3.62	0.0177	>25	>6.9
Ciclesonide ^#,†^	Alvesco, others	Glucocorticoid ligand	Anti-inflammatory agent	4.07	0.4907	>25 ^§^	>6.1
Benidipine hydrochloride ^#^	Coniel	Calcium channel blocker	Cardiovascular agent	4.07	0.7234	>25	>6.1

^1^ DrugBank database (version 5.0) was used for characterizing FDA-approved drugs; ^2^ 50% inhibitory concentration (IC_50_); ^3^ standard deviation (SD) of replicated IC_50_ values; ^4^ 50% cytotoxicity concentration (CC_50_); ^5^ therapeutic index (TI): ratio of CC_50_/IC_50_; ^#^ drug acting on the early stage of the viral life cycle, according to time-of-addition study; * drug acting on the late stage of the viral life cycle, according to time-of-addition study; ^†^ activity in SARS-CoV-2 system [32]; ^§^ CC_50_ > 50 μM in Vero cells [32].

**Table 4:** Hit profiling and anti-MERS-CoV efficacies of selected bioactives in Vero cells ^1^.

Inhibitor Name	Pharmaceutical Action	IC_50_^2^ (μM)	SD ^3^ (±)	CC_50_^4^ (μM)	TI ^5^
Emetine dihydrochloride	Anti-neoplastic agent	0.08	0.0054	>25	>312.5
Oxyclozanide	Anti-parasitic agent	0.07	0.0060	20.92	298.9
Cycloheximide	Protein synthesis inhibitor	0.16	0.0140	>25	>156.3
Lanatoside C	Cardiotonic agent	0.19	0.0103	>25	>131.6
Calcimycin	Antibacterial agent	0.20	0.0165	18.10	90.5
Digitoxigenin	Cardiotonic agent	0.29	0.0220	>25	>86.2

^1^ DrugBank database (version 5.0) was used for characterizing bioactives; ^2^ 50% inhibitory concentration (IC_50_); ^3^ standard deviation (SD) of replicated IC_50_ values; ^4^ 50% cytotoxicity concentration (CC_50_); ^5^ therapeutic index (TI): ratio of CC_50_/IC_50_.

## Data Availability

The data presented in this study are available on request from the corresponding author.

## Supplementary Materials

The following are available online at https://www.mdpi.com/article/10.3390/v13040651/s1. Figure S1: Example images of MERS-CoV inhibition in Vero cells, Figure S2: Time-of-addition study with additional FDA-approved drugs, Figure S3: Structural relationship between inhibitors, Table S1: Inhibitors identified by HCS with SI > 6.
