# Supplementary material for: Screening of FDA-Approved Drugs Using a MERS-CoV Clinical Isolate from South Korea Identifies Potential Therapeutic Options for COVID-19
Source: Viruses. 2021 Apr 9;13(4):651. doi: 10.3390/v13040651 (PMC8069929; doi:10.3390/v13040651)
Supplement: Supplementary file 1 [file viruses-13-00651-s001.pdf]

Supplementary Information

Screening of FDA-Approved Drugs Using a MERS-CoV Clinical Isolate from South Korea Identifies Potential Therapeutic Options for COVID-19

Meehyun Ko [1,†], So Young Chang [1,†], Soo Young Byun [2], Aleksandr Ianevski [3], Inhee Choi [4], Anne-Laure Pham Hung d'Alexandry d'Orengiani [1], Erlend Ravlo [3], Wei Wang [3], Magnar Bjørås [3], Denis E. Kainov [3,5,6], David Shum [2], Ji-Young Min [1,‡,*] and Marc P. Windisch [7,8,*]

Supplementary Material and Methods

1.1. Reagents

Chloroquine diphosphate (CQ; C6628), chlorpromazine hydrochloride (CPZ; C8138), and cyclosporine A (CsA; C1832) were purchased from Sigma-Aldrich (St. Louis, MO, USA). Lopinavir (LPV; S1380) was purchased from SelleckChem (Houston, TX, USA). Chloroquine was dissolved in Dulbecco's phosphate-buffered saline (DPBS; Welgene, Gyeongsan, Republic of Korea), and other reagents were dissolved in DMSO for the screening. Anti-MERS-CoV S antibody was purchased from Sino Biological Inc. (Beijing, China). Alexa Fluor 488 goat anti-rabbit IgG (H+L) secondary antibody and Hoechst 33342 were purchased from Molecular Probes/Thermo Fisher Scientific. Paraformaldehyde (PFA) (32% aqueous solution) and normal goat serum were purchased from Electron Microscopy Sciences (Hatfield, PA, USA) and Vector Laboratories, Inc. (Burlingame, CA, USA), respectively.

1.2. Time-of-Addition Assays

Twelve FDA-approved drugs were selected after DRC analysis for time-of-addition assays to characterize the mechanism of action. Briefly, Vero cells were seeded at 1.2×10^4 cells per well in Opti-PRO™ SFM supplemented with 4 mM L-glutamine and 1× antibiotic-antimycotic solution in black, 96-well, µClear plates (Greiner Bio-One; Kremsmünster, Austria) at 24 h prior to the experiment. Subsequently, cells were infected with MERS-CoV/KOR/2015 at an MOI of 5 at 4 °C for 1 h to synchronize the infection, followed by three washes with Dulbecco's phosphate-buffered saline (DPBS), and transferred to a 37˚C incubator. Drugs were added to cells at various time points pre- or post-infection; 1 h prior to or at 0, 1, 2, 3, or 4 hpi. Drugs were added to infected cells at a concentration of 1, 5, or 10 µM depending on the IC_{90} value of the compound. Chloroquine, which was used as an early-stage inhibitor control, was used at a concentration of 100 µM. Cells were fixed with 4% PFA at 6 hpi. IFA and image analyses were performed as described.

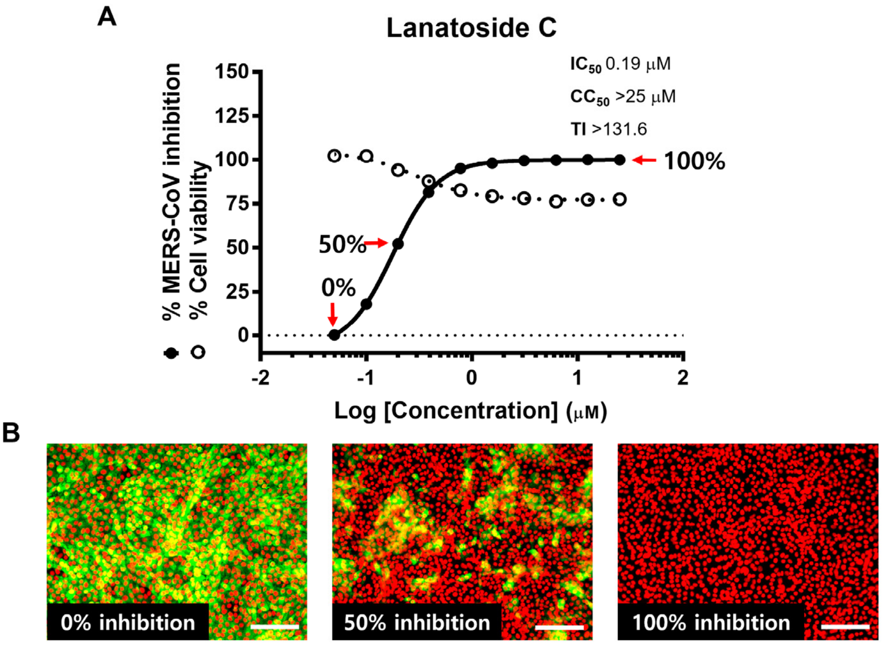

Figure S1. Example images of MERS-CoV inhibition in Vero cells. (**A**) A representative dose-response curve (DRC) for lanatoside C inhibition of MERS-CoV in Vero cells. (**B**) HCS was performed using an image-based assay, and compound efficacy was measured by inhibition of MERS-CoV S protein expression. Images depict 0%, 50%, and 100% inhibition, as indicated in the DRC. Scale bars, 100 μm.

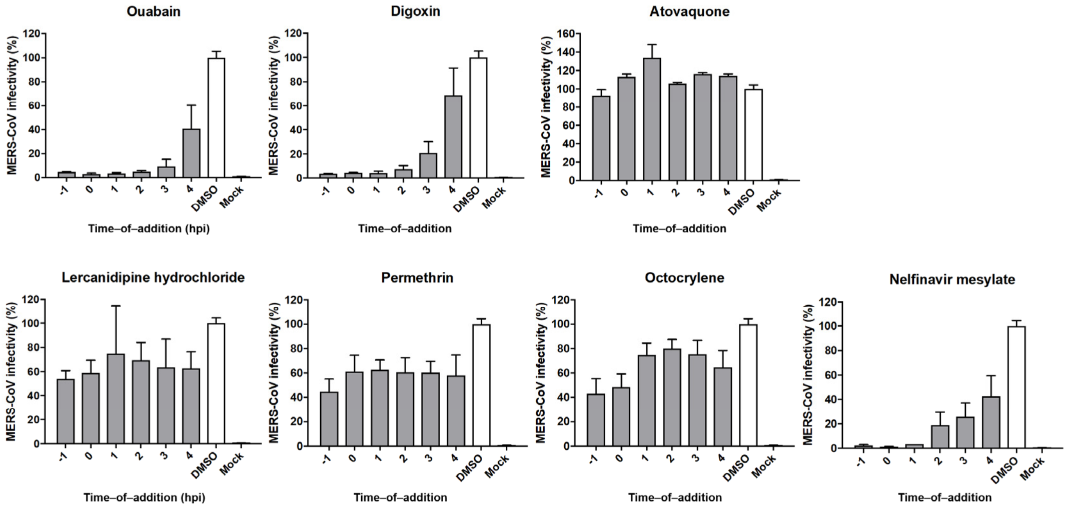

Figure S2. Time-of-addition study with additional FDA-approved drugs. Seven FDA-approved drugs not shown in Figure. 2 were analyzed by time-of-addition experiments. Vero cells were infected with MERS-CoV at a multiplicity of infection of 5, and drugs were administered at six-time points pre- or post-infection as indicated. Drugs were used at concentrations above their 90% inhibitory concentration (IC_{90}) values.

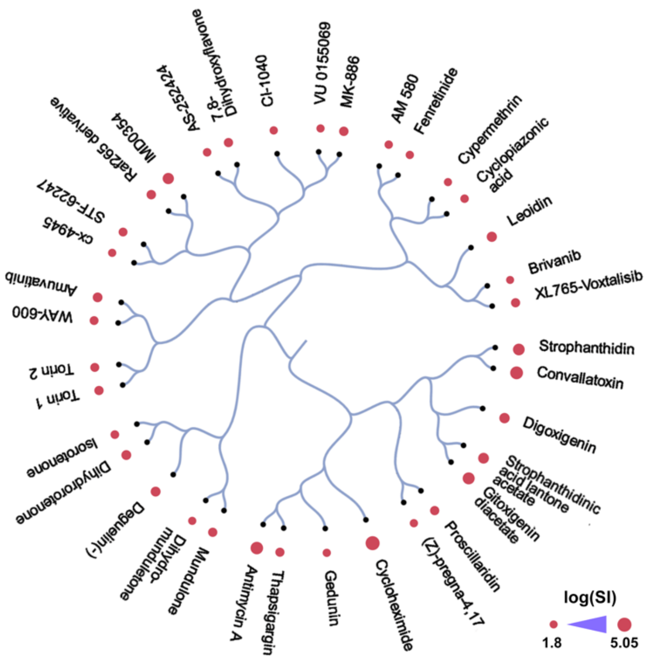

Figure S3. Structural relationship between inhibitors**.** Dendrogram showing the structural relationship of 36 selected inhibitors with anti-MERS-CoV activity. Compounds were clustered based on their structural similarity calculated by ECPF4 fingerprints and the Tanimoto coefficient [36]. The activity of the compounds was quantified using the log SI values and shown as bubbles. Bubble size corresponds to compounds' log TI and SI (https://cspade.fimm.fi/).

Table S1. Inhibitors identified by HCS with SI >6.

Inhibitor Name	IC_{50} [1]	CC_{50} [2]	SI [3]
Cycloheximide	0.16	>25	>156.3
Convallatoxin	0.31	>25	>80.6
Gitoxigenin diacetate	0.48	>25	>52.1
Antimycin A	0.36	>25	>69.4
Strophanthidinic acid lantone acetate	0.56	>25	>44.6
Strophanthidin	0.56	>25	>44.6
IMD0354	0.25	8.74	35.0
Digoxigenin	1.13	>25	>22.1
Leoidin	1.26	>25	>19.8
Deguelin(-)	1.47	>25	>17.0
Dihydrorotenone	1.52	>25	>16.4
Amuvatinib (MP-470)	1.60	>25	>15.6
Raf265 derivative	1.86	>25	>13.4
MK-886	1.91	>25	>13.1
Proscillaridin	2.05	>25	>12.1
Torin 1	2.08	>25	>12.0
Mundulone	1.21	14.58	12.0
7,8-Diydroxyflavone	2.11	>25	>11.8
Voxtalisib (XL765)	2.17	>25	>11.5
Thapsigargin	0.49	5.55	11.3
Torin 2	2.44	>25	>10.2
STF-62247	2.54	>25	>9.8
WAY-600	2.58	>25	>9.7
Isorotenone	2.90	>25	>8.6

Cyclopiazonic acid	3.17	>25	>7.9
AS-252424	1.78	14.14	7.9
AM 580	3.32	>25	>7.5
CI-1040	3.50	>25	>7.1
Fenretinide	2.80	19.85	7.1
Gedunin	3.59	>25	>7.0
Silmitasertib (cx-4945)	3.66	>25	>6.8
VU 0155069	3.69	>25	>6.8
Dihydro-munduletone	3.72	>25	>6.7
Cypermethrin	3.77	>25	>6.6
Guggulsterone	3.85	>25	>6.5
Brivanib (BMS-540215)	4.13	>25	>6.1

[1] 50% inhibitory concentration (IC_{50}); [2] 50% cytotoxicity concentration (CC_{50});
[3] Selectivity Index (SI): ratio of CC_{50}/IC_{50}.
